# Regulation of Cathepsin E gene expression by the transcription factor Kaiso in MRL/lpr mice derived CD4+ T cells

**DOI:** 10.1038/s41598-019-38809-y

**Published:** 2019-02-28

**Authors:** Sumie Hiramatsu, Katsue S Watanabe, Sonia Zeggar, Yosuke Asano, Yoshia Miyawaki, Yuriko Yamamura, Eri Katsuyama, Takayuki Katsuyama, Haruki Watanabe, Mariko Takano-Narazaki, Yoshinori Matsumoto, Tomoko Kawabata, Ken-Ei Sada, Jun Wada

**Affiliations:** Department of Nephrology, Rheumatology, Endocrinology and Metabolism, Okayama University Graduate School of Medicine, Dentistry and Pharmaceutical Sciences, Okayama, Okayama, 700-8558 Japan

## Abstract

Global DNA hypomethylation in CD4+ cells in systemic lupus erythematosus (SLE) was suggested to play a key role in the pathogenesis. To identify new methylation-sensitive genes, we integrated genome-wide DNA methylation and mRNA profiling data in CD4+ cells of MRL/lpr (MRL) and C57BL6/J (B6) mice. We identified Cathepsin E (*Ctse*), in which 13 methyl-CpGs within 583 bp region of intron 1 were hypomethylated, and *Ctse* mRNA upregulated in MRL compared with B6 mice. One of methyl-CpGs, mCGCG was 93.3 ± 2.05% methylated in B6 mice, while 80.0 ± 6.2% methylated and mutated to CGGG in MRL mice. Kaiso is known to bind to mCGCG and we hypothesized that it represses expression of *Ctse* in B6 mice. The binding of Kaiso to mCGCG site in B6 mice was reduced in MRL mice revealed by ChIP-PCR. EL4 cells treated with 5-azaC and/or Trichostatin A showed the suppression of binding of Kaiso to mCGCG motif by ChIP-PCR and the overexpression of *Ctse* was demonstrated by qPCR. *Ctse* gene silencing by siRNA in EL4 cells resulted in reduction of IL-10 secretion. The hypomethylation of mCGCG motif, reduced recruitment of Kaiso, and increased expression of *Ctse* and *Il-10* in CD4+ cells may be involved in the pathogenesis of SLE.

## Introduction

Systemic lupus erythematosus (SLE) is a systemic autoimmune disease provoked by aberrant immune responses directed against cells and tissues, resulting in inflammation and organ damage^1^. Five-year survival in patients with SLE has improved from 50% in the 1950 s to over 90% currently^2^. However, the early diagnosis of the disease is still challenging and the mortality remains high compared with the general population. Although genome-wide association studies (GWAS) have supported the importance of genetic background for development of SLE^3^, incomplete concordance in monozygotic twins who carry the same SLE-susceptibility genes suggests that environmental and epigenetic factors are also important for its pathogenesis^4^. Epigenetic processes refer to heritable modifications that regulate gene expression and affect cellular functions without any changes in the genomic sequence. DNA methylation, histone modification, and altered miRNA profiling are widely recognized as the key epigenetic mechanisms. DNA methylation occurs on the carbon 5 position of the pyrimidine ring of cytosine residues from CpG dinucleotides, although it was recently observed to occur on other motifs, CHG or CHH (H = A, C, T), in embryonic tissue and induced pluripotent stem cells^5^. In general, methylation on genomic DNA represses gene expression, while demethylation is associated with enhanced transcriptional activities. The methylation status is critically involved in the transcriptional regulation by altering the accessibility of several transcription factors to the targeted promoters, genome imprinting, and X-chromosome inactivation. The series of evidence, such as DNA hypomethylation in SLE CD4+ T cells^6^, ultraviolet light and drug-induced DNA hypomethylation^7,8^, and association of disease activity with DNA hypomethylation^4^ suggested the epigenetic mechanisms in the development of lupus. Therefore, study of epigenetic mechanisms may provide important clues how environmental factors contribute to the phenotypic expression of autoimmunity related diseases.

We previously demonstrated that hypomethylation of a CpG within cAMP response element (CRE) motif links to increased expression of PP2Acα in T cells derived from the patients with SLE^9^. We also performed global miRNA and mRNA profiling in CD4+ T cells purified from spleen of MRL/lpr lupus-prone mice (MRL) and compared with the C57BL/6 (B6) and isolated miR-200a-3p, which is involved in the hypoproduction of IL-2 in T cells by targeting CtBP2 complex^10^. To identify the putative methylation-sensitive genes involved in the pathogenesis of SLE, we performed the integration analysis of genome-wide DNA methylation and global mRNA profiling in CD4+ T cells purified from spleen of MRL and compared with B6 mice. During the screening, we have identified cathepsin E (*Ctse*) gene, in which 13 methyl-CpG-dinucleotides located within 583 bp region of intron 1 were hypomethylated, and *Ctse* mRNA was highly expressed in MRL mice compared with B6 mice. One of 13 methyl-CpGs, methyl-CGCG (mCGCG) in B6 mice was hypomethylated as well as mutated to CGGG in MRL mice. Kaiso (ZBTB33; zinc finger and BTB domain) is a member of to the BTB (BR-C, ttk, and bab)/POZ (Pox virus and zinc finger) family, and reported to bind to DNA with dual-specificity in both a sequence- (Kaiso-binding site; CTGCNA) and methyl-CpG (mCGCG) specific manner via C2H2 zinc finger (ZF)^11^ and methyl-DNA-binding (MBD) domains^12^, respectively.

Here, we demonstrate that Kaiso directly binds to mCGCG site in intron 1 of *Ctse* gene in methyl-CpG-dependent manner and represses the transcriptional activity of *Ctse* in B6 mice, while the demethylation and mutation of mCGCG to CGGG caused the reduced binding of Kaiso and up-regulated expression of *Ctse*. In immune-mediated cells, *Ctse* was shown to be involved in processing of antigenic peptides during MHC class II-mediated antigen presentation in dendritic cells and macrophages^13^. In contrast, the role of *Ctse* in T cells in normal physiology and pathobiology in autoimmune diseases remains unexplored. We also found that knockdown of *Ctse* gene in cultured EL4 cells resulted in decreased production of IL-10 and that up-regulated expression of IL-10 was observed in CD4+ T cells isolated from MRL mice compared with B6 mice. IL-10 is known to be elevated in the serum and tissues of patients with systemic lupus erythematosus (SLE) and the expression of IL-10 is activated by Stat3 (signal transducer and activator of transcription 3) in peripheral CD4+ T cells^14^. Because of its B cell-promoting effects, T cell-derived IL-10 may contribute to autoantibody production and tissue damage in SLE. In this communication, we postulate that *Ctse* represents a new methylation-sensitive gene, like previously recognized CD70, CD40L and CD11a^15^, contributes to the pathogenesis of SLE.

## Results

### *Ctse* gene is hypomethylated and upregulated in CD4+ T cells of MRL mice

**Figure 1:**
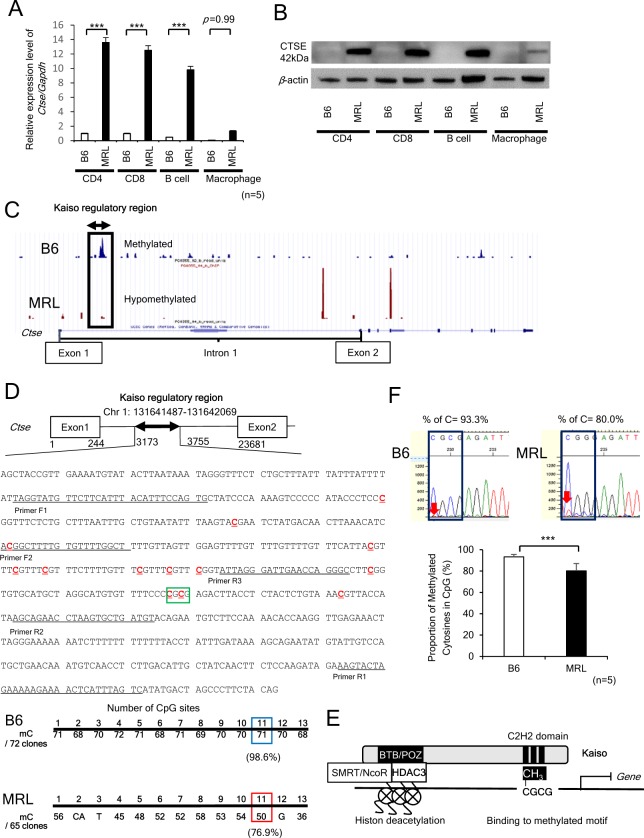
The expression and methylation status of *Ctse* gene in CD4+ T cells isolated from MRL/lpr lupus-prone (MRL) and C57BL/6 (B6) mice. (**A**) mRNA expression of *Ctse* in CD4+, CD8+, B cells and macrophages in B6 and MRL mice. ***p < 0.001. (**B**) Western blot analyses of CTSE in CD4+, CD8+, B cells and macrophages in B6, MRL and mice. (**C**) Methylated region expanding 583 bp in *Ctse* intron 1 in B6 mice demonstrated by UCSC Genome Browser. In MRL mice, 583 bp region is hypomethylated. Duplicate reads are indicated by arrows. (**D**) Nucleotide sequence within 583 bp flanking region of *Ctse* intron 1 in B6 mice. Among 13 CpGs (red and underlined), 11th CpG (boxed) includes mCGCG binding motif for Kaiso. After genomic DNAs were bisulphite converted, amplified by PCR using Primer F1 and R1, subcloned, and isolated clones sequenced. 98.6% of CpGs (71 out of 72 colonies) in B6 mice and 76.9% CpGs (50 of 65) in MRL mice were methylated. (**E**) The structure of Kaiso, SMRT (Silencing Mediator of Retinoic acid and Thyroid hormone receptors)/N-CoR (Nuclear hormone receptor Co-Repressor), and HDAC3 (Histone deacetylase 3) complex. BTB (for BR-C, ttk and bab), POZ (for Pox virus and Zinc finger) and C2H2 zinc-finger domains are shown. (**F**) DNA sequencing of bisulphite converted DNAs in *Ctse* intron 1 in B6 and MRL mice. Percentage of methylation of 11th CpG is shown (n = 5). ***p < 0.001.

To identify novel methylation-sensitive genes in SLE, we integrated genome-wide DNA methylation and mRNA profiling data in splenic CD4+ T cells isolated from MRL and compared with B6 mice. The differentially expressed 188 genes with statistical significance revealed by Cuffdiff program were merged with 1,557 enriched regions with significant methylation peaks demonstrated by MACS program. The 7 genes were identified and they were associated with significant methylation peaks in B6 mice but not in MRL mice (Supplementary Table 1). Since RNA sequence data demonstrated most abundant and highly up-regulated expression and of *Ctse* in splenic CD4+ T cells from MRL mice, we next investigated the expression of *Ctse* in immune-mediated cells. The mRNA and protein levels of *Ctse* prominently were increased in splenic CD4+ T cells from MRL compared with those from B6 (Fig. 1A,B, and Supplementary Fig. 1). Although *Ctse* was shown to be involved in processing of antigenic peptides during MHC class II-mediated antigen presentation in macrophages, the expression was prominently up-regulated in CD4+ and CD8+ T cells, and B cells in MRL mice, but it is not the case with macrophages (Fig. 1A,B).

In genome-wide DNA methylation analysis, the methylated region expanded 583 bp in intron 1 of *Ctse* in B6 mice was identified by MACS program, while it was hypomethylated in MRL mice (Supplementary Table 1 and Fig. 1C). Two peaks observed in MRL mice were excluded as duplicate reads by MACS program (Fig. 1C). Next, we searched the specific methylated CpGs by bisulphite sequencing in this region. From B6 (n = 1) and MRL (n = 1) mice, genomic DNAs were isolated, bisulphite converted, 440 bp region including 13 CpGs in intron 1 of *Ctse* amplified by PCR, cloned, and sequenced. As shown in Fig. 1D, 2 CpGs were mutated, while 11 CpGs were hypomethylated in MRL compared with B6. We searched candidate transcription factors which bind to the 583 bp methylated region in intron 1 through JASPAR database. Kaiso was found and it is known to specifically recognize the methylated DNA motif (mCGCG) through the C2H2 zinc-finger domains, recruits the SMRT (Silencing Mediator of Retinoic acid and Thyroid hormone receptors)/N-CoR (Nuclear hormone receptor Co-Repressor)/HDAC3 (Histone deacetylase 3) complex, leading to suppression of its target gene^16^ (Fig. 1E). Indeed, mCGCG motif at 11^th^ CpG site (Fig. 1D, boxed) demonstrated to be mutated to CGGG and 21.7% reduction of DNA methylation in MRL (76.9%) compared to B6 mice (98.6%). Furthermore, bisulphite converted genomic DNAs from B6 (n = 5) and MRL (n = 5) were sequenced to confirm the difference of DNA methylation pattern between B6 and MRL. The 11^th^ CpG was hypomethyalted in MRL (80.0 ± 6.2%) compared with B6 (93.3 ± 2.05%) (p = 3.82 × 10^−5^) (Fig. 1D).

### The binding of Kaiso to mCGCG was reduced by hypomethylation

**Figure 2:**
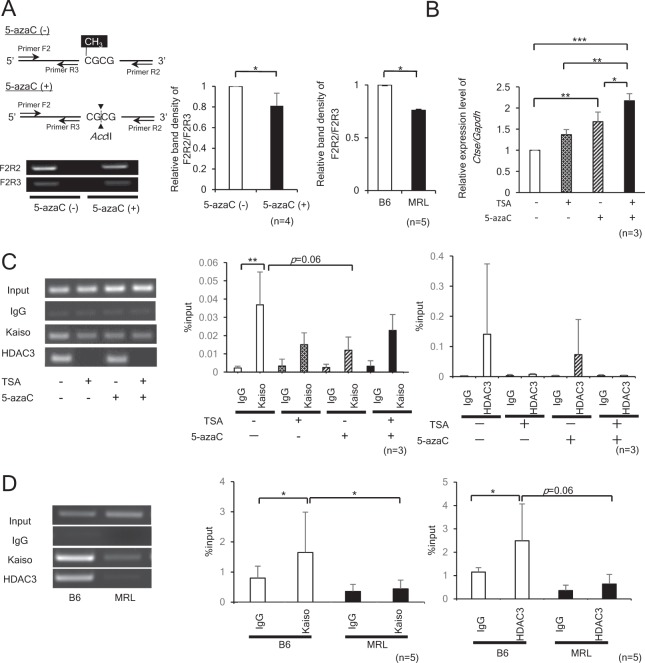
Kaiso binding to mCGCG motif located in *Ctse* intron 1 and HDAC3 (Histone deacetylase 3) in CD4+ T cells from MRL mice. (**A**) Methylation specific PCR analysis in EL4 cells treated with or without 1 μM of 5-azaC, and CD4+ T cells derived from B6 and MRL mice. Methylation-sensitive restriction enzyme *Acc*II digests unmethylated CGCG motifs, while methylated CGCG is resistant to *Acc*II. The genomic DNAs were digested with *Acc*II, amplified by PCR using primer sets; Primer F2/R2 and Primer F2/R3. The densitometry intensity ratios of PCR products (F2R2/F2R3) are shown. *p < 0.05. (**B**) mRNA expression of *Ctse* in EL4 cells treated with 1 μM of 5-azacytidine (5-azaC) and/or 100 nM of Trichostatin A (TSA). *p < 0.05, **p < 0.01, ***p < 0.001. (**C**) DNA binding of Kaiso and HDAC3 around mCGCG motif located in *Ctse* intron 1 examined using ChIP-PCR in EL4 cells. EL4 cells treated with 1 μM of 5-azacytidine (5-azaC) and/or 30 nM of Trichostatin A (TSA). ChIP-PCR was performed in 3 independent culture experiments. **p < 0.01. (**D**) DNA binding of Kaiso and HDAC3 around mCGCG motif in CD4+ T cells isolated from B6 and MRL mice. The amount of precipitated DNAs was quantified using real-time PCR with primers specific to 583 bp region in *Ctse* intron 1. ChIP-PCR was performed in 3 independent animals. *p < 0.05.

To determine the impacts of hypomethylation of mCGCG motif on the transcriptional regulation of *Ctse*, we induced DNA hypomethylation in EL4 cells using well-known DNA methylation inhibitor, 5-azaC. The genomic DNA was purified from EL4 cells treated with or without 1 μM of 5-azaC for 48 hours and incubated with the methylation-sensitive restriction enzyme *Acc*II, which digests only unmethylated CGCG motifs. By the treatment of 5-azaC, the intensity of PCR products derived from *Acc*II-resistant mCGCG was reduced, while those from *Acc*II-sensitive unmethylated CGCG unaltered (Fig. 2A, Supplementary Fig. 1). Next, EL4 cells were treated with 5-azaC and/or histone deacetylase (HDAC) inhibitor, Trichostatin A (TSA). The levels of *Ctse* mRNA expression were up-regulated under the treatment of 5-azaC or TSA in EL4 cells and we also observed the additive effects under simultaneous treatment of 5-azaC and TSA (Fig. 2B). Since TSA inhibits HDACs 1, 3, 4, 6, and 10, and it is not specific only for HDAC3, we next performed ChIP-qPCR to check the binding of Kaiso and HDAC3 to CGCG motif in EL4 cells. Under the treatment of 5-azaC and TSA in EL4 cells, ChIP-qPCR demonstrated that the bindings of Kaiso and HDAC3 to the hypomethylated CGCG motif seemed to be reduced without statistical differences (Fig. 2C, Supplementary Fig. 1). Since the treatment of EL4 cells with 5-azaC results in global hypomethylation, we cannot negate the possibility that the increase in *Ctse* expression is caused by hypomethylation of other regions of genomic DNA. Thus, we next compared *Acc*II-resistant mCGCG and *Acc*II-sensitive unmethylated CGCG in CD4+ T cells derived from B6 and MRL mice. The ratio of *Acc*II-resistant mCGCG was reduced in MRL mice (Fig. 2A). In addition, the bindings of Kaiso and HDAC3 to the *Ctse* intron 1 in CD4+ T cells of MRL and B6 mice. ChIP-qPCR revealed that Kaiso and HDAC3 bindings to the *Ctse* intron 1 were reduced in MRL CD4+ T cells compared with B6 CD4+ T cells (Fig. 2D, Supplementary Fig. 1).

### Binding of Kaiso to Kaiso regulatory region of *Ctse* gene

**Figure 3:**
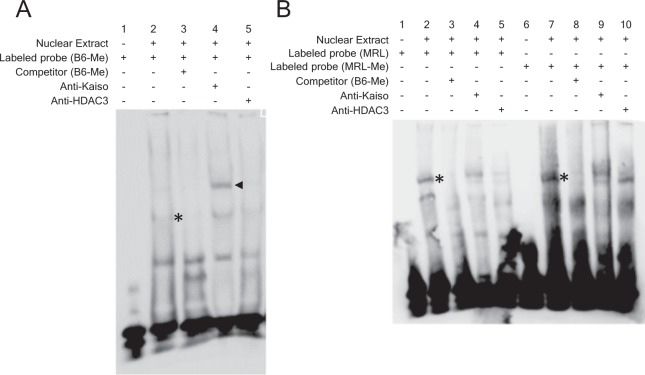
The binding of Kaiso on the mCGCG motif of Kaiso regulatory region of *Ctse* gene. (**A**) Biotin-labeled and methylated probe (B6-Me) was incubated with 28 μg of nuclear protein from EL4 cells, and putative Kaiso and DNA complexes is indicated by asterisk in lane 2. The formation of Kaiso and DNA complexes was inhibited by excess amounts of unlabeled competitor (lane 3). The Kaiso and DNA complexes demonstrated supershift by the addition of 4 μg of anti-Kaiso Ab (arrow head in lane 4). (**B**) Biotin-labeled, unmethylated (MRL), and methylated (MRL-Me) probes were incubated with 28 μg of nuclear protein from EL4 cells. The putative Kaiso and DNA complexes are indicated by asterisks in lanes 2 and 7 and they reveal no supershift with anti-Kaiso Ab and anti-HDAC3 Ab.

To demonstrate the binding of Kaiso to Kaiso regulatory region of *Ctse* gene, we incubated nuclear proteins from EL-4 cells with labeled double-stranded oligonucleotides containing the CGCG motif (Supplementary Table 2). In EMSA (Fig. 3A), the biotin-labeled methylated wild type probe (B6-Me; mCGCG) formed DNA-protein complex (asterisk), which were supershifted by anti-Kaiso antibody (arrow head), and disappeared by cold competitor (B6-ME). Anti-HDAC3 antibody failed to demonstrate the supershift. The incubation of biotin-labeled MRL-Me (mCGGG) or MRL (CGGG) probes with nuclear extracts demonstrated the presence of DNA-protein complexes (asterisks) and they were disappeared by cold competitor (B6-Me), which efficiently binds to Kaiso. However, they were not efficiently supershifted by anti-Kaiso and anti-HDAC3 antibodies (Fig. 3B), suggesting reduction of the binding of Kaiso to the Kaiso regulatory region of *Ctse* gene in MRL mice.

### Kaiso regulates the *Ctse* promoter activity

**Figure 4:**
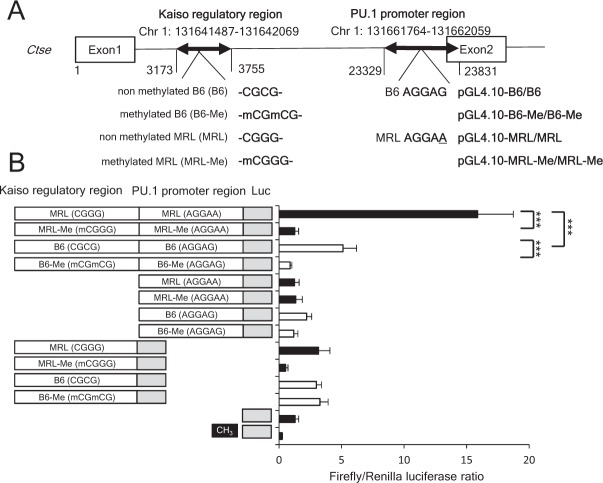
Luciferase assay of Kaiso regulatory region of the *Ctse* gene. (**A**) Nucleotide sequences of CGCG motif in Kaiso regulatory region expanding chromosome 1: 131641487–131642069, and AGGAG motif in PU.1 promoter region expanding chromosome 1: 131661764–131662059 in prepared pGL4.10 [*luc2*] Vector constructs. (**B**) pGL4.10 [*luc2*] Vector constructs (1 μg) were transfected into EL4 cells with pRL-TK plasmid and Firefly/Renilla ratio of pGL4.10 (empty vector) was set as 1.0. MRL/MRL construct showed significantly higher luciferase activity compared with pGL4.10-B6/B6 construct (***p < 0.001). DNA methylation of pGL4.10-MRL/MRL and pGL4.10-B6/B6 constructs significantly reduced luciferase activity indicated as pGL4.10-MRL-Me/MRL-Me and pGL4.10-B6-Me/B6-Me constructs, respectively (***p < 0.001).

**Figure 5:**
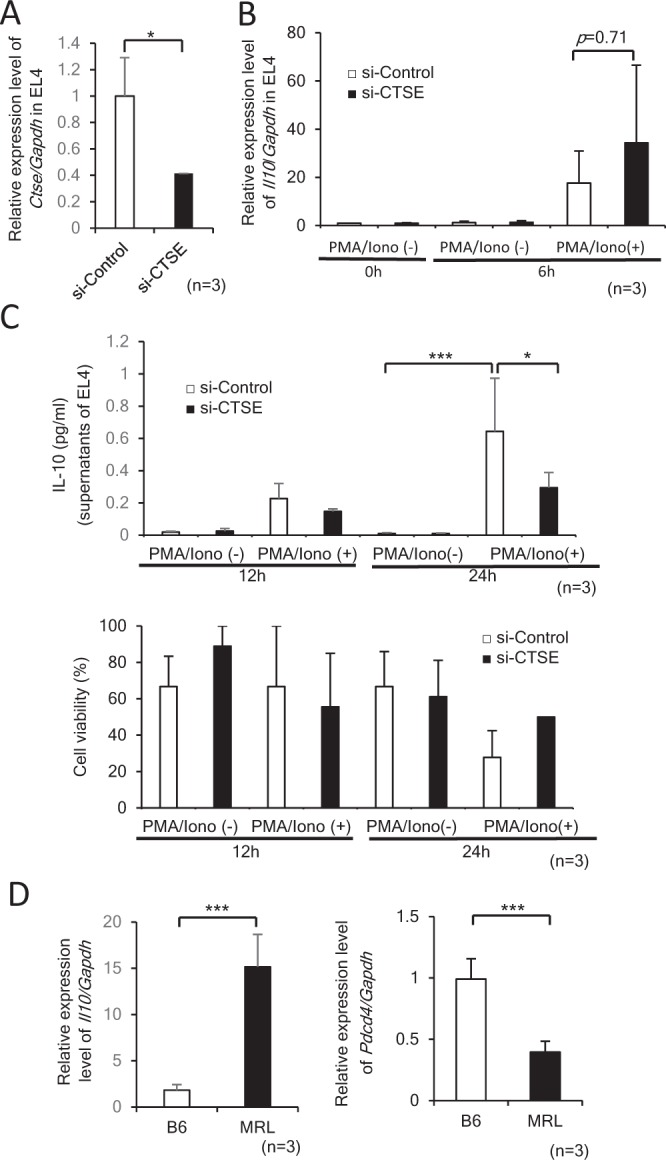
Expression of *Il10* in EL4 cells transfected with siRNA for *Ctse* and CD4+ T cells isolated from B6 and MRL mice. (**A**) The knockdown and mRNA expression of *Ctse* in EL4 cells transfected with 5 μM control siRNA (si-Control) or *Ctse* siRNA (si-CTSE). *p < 0.05. (**B**) mRNA expression of *Il10* in EL4 cells transfected with siRNAs for 24 hours and subsequently stimulated with 10 ng/ml phorbol 12-myristate 13-acetate (PMA) and 1 μM Ionomycin for 6 hours. (**C**) The concentration of IL-10 in supernatants and cell viability of cultured EL4 cells. EL4 cells were transfected with indicated siRNAs for 24 hours, stimulated with 10 ng/ml PMA and 1 μM Ionomycin for 12 or 24 hours. In cell viability, percentage of Trypan Blue negative cells is shown. *p < 0.05, ***p < 0.001. (**D**) mRNA expression of *Il10* and *Pdcd4* in CD4+ T cells isolated from B6 and MRL mice. ***p < 0.001.

To demonstrate transcriptional repression activity of Kaiso, we subcloned the Kaiso regulatory region with CGCG motif and PU.1 promoter region with PU.1 binding site into pGL4.10 [*luc2*] Vector. PU.1 promoter region was known to display the most potent promoter activity of *Ctse*^17^. The various constructs are shown in Fig. 4A and Supplementary Fig. 2. pGL4.10-MRL/MRL construct demonstrated the highest luciferase activity, while pGL4.10-B6/B6 construct revealed significantly lower luciferase activity (Fig. 4B). It suggested that CGCG motif at Kaiso regulatory region is critical for the repression of transcriptional activity of *Ctse* gene. We next investigated the effect of DNA methylation of Kaiso regulatory region. The methylation of constructs significantly and prominently repressed luciferase activities in pGL4.10-MRL-Me/MRL-Me and pGL4.10-B6-Me/B6-Me constructs compared with corresponding unmethylated constructs (Fig. 5B). Taken together, Kaiso bind to the mCGCG motif in Kaiso regulatory region and repress its transcriptional activity.

### *Ctse* knockdown in EL4 cells reduces IL-10 secretion in EL4 cells

The treatment of EL4 cells by si-CTSE resulted in the 60% reduction of mRNA expression of *Ctse* compared with si-Control (negative control) treated cells (Fig. 5A). Although the reduction of *Ctse* did not significantly alter the mRNA expression of *Il10* after the stimulation with PMA/Iono (Fig. 5B), the secretion of IL-10 into the culture media was significantly reduced at 24 hours after the stimulation with PMA/Iono (Fig. 5C), suggesting suppressed secretion of IL-10 was not caused by the reduction of transcriptional activity of *Il10*. The viability of EL4 was investigated by Trypan Blue and the percentage of live cells are similar in both si-Control and si-CTSE groups (Fig. 5C). The upregulation of *Ctse* in CD4+ T cells in MRL mice was associated with the upregulation of transcriptional activity of *Il10* (Fig. 5D). The upregulated expression of *Il10* under the control of *Ctse* was specific phenomenon by the treatment of si-CTSE and it was down-regulated in CD4+ T cells from MRL mice (Fig. 5B,D). The tumor suppressor, programed cell death 4 (*Pdcd4*), inhibits the translation of *Il10* transcripts and the secretion is enhanced in splenocytes in *Pdcd4* knockout mice^18^. As shown in Fig. 5D, mRNA expression of *Pdcd4* in CD4+ T cells was higher in B6 mice compared to MRL mice, as previously reported^19^. Taken together, the hypomethylation and mutation of a Kaiso-binding site in the *Ctse* intron 1 prevents the biding of Kaiso-HDAC3 complex, which may link to the increased histone acetylation, upregulated mRNA expression of *Ctse* gene, and enhanced secretion of IL-10 from CD4+ T cells in MRL mice (Supplementary Fig. 3).

### *CTSE* and *IL10* mRNA were elevated in CD4+ T cells from the patients with SLE

**Figure 6:**
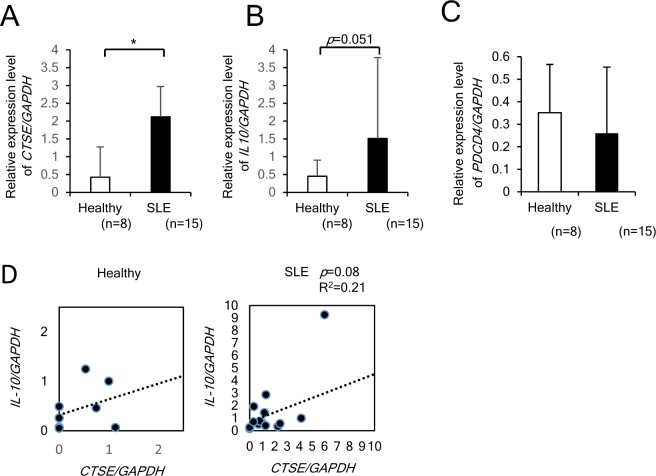
The increased levels of *CTSE* and *IL10* transcripts in CD4+ T cells from SLE patients. (**A**) and (**B**) mRNA expression of *CTSE* and *IL10* in CD4+ T cells isolated from healthy (n = 8) and the patients with SLE (n = 15). (**C**) mRNA expression of *PDCD4*. There is no significant difference in healthy subjects and patients with SLE. (**D**) The simple correlation between the *CTSE* and *IL10* transcript levels. *p < 0.05.

We investigated the expression levels of *CTSE* and *IL10* mRNAs in circulating CD4+ T cells isolated from SLE patients and healthy subjects. The demographics of enrolled SLE patients are shown in Supplementary Table 3. SLE patients also showed the similar tendency with MRL mice; the expression level of *Ctse* and *IL10* mRNAs in CD4+ T cells is upregulated in lupus patients than healthy control (Fig. 6A,B), while PDCD4 mRNA lower tendency in lupus patients than healthy control (Fig. 6C). However, there was no significant correlation between *Ctse* and *IL10* mRNA expression levels (Fig. 6D). Although we searched the correlation with clinical parameters appeared in Supplementary Table 3, we failed to demonstrate the correlation of *Ctse* and *IL10* mRNA expression levels with any clinical parameters.

## Discussion

Methylation of target sequences prevents the binding of certain transcriptional activators, such as AP-2, thereby suppressing gene expression^20^. Methyl-CpG-binding proteins (MBPs) are able to prevent the binding of transcription factors and suppress gene expression from a distance^21^, and it also attracts chromatin remodeling complexes that modify adjacent histones, resulting in a condensed nucleosome structure, making the locus inaccessible to transcription factors^22^. Methylated DNA can be recognized by MBPs, which belong to three different structural families in mammals: the MBD family, Kaiso-like proteins, and the SRA (SET and RING finger associated domain) proteins^23^. They are involved in many biological and pathological processes, including control of genome stability, early embryonic development, neuron maturation, and T cell differentiation, as well as human diseases such as Rett syndrome and cancer^24^. The transcription factors which regulate the expression of *Ctse* had been reported. Cook *et al*. reported that the expression of the *Ctse* gene is probably dependent upon the balance between positive-acting, tissue-specific transcription factors such as GATA1 and PU1, and the negative influence of the ubiquitous factor YY1^25^. Okamoto *et al*. identified the several unique transcriptional activators, such as Sp1, AP-1 and cEts-1 for the mouse *Ctse* gene^17^. In current investigation, we present the first evidence that *Ctse* as a newly identified methylation-sensitive gene, which is involved in the pathogenesis of SLE. Specifically, CD4+ T cells from MRL mice demonstrated decreased binding of the transcriptional factor Kaiso to Kaiso regulatory motif at intron 1 of *Ctse* gene because of its lower methylation status and point mutation. Importantly, upregulated *Ctse* expression level in T cells correlated with the IL-10 expression in EL4 cells and CD4+ cells from MRL mice. The upregulation of IL-10 in lupus T cells is well-known^14^ and *Pdcd4*, the know suppressor of IL-10 production^26^, is reported to be down-regulated in lupus T cells^19^. In EL4 cells and CD4+ cells from MRL mice, increased production of IL-10 and reduced expression of *Pdcd4* were observed. Although the precise molecular mechanism remains unknown, the knockdown of *Ctse* gene resulted in reduced IL-10 release.

Among various cathepsins, it has been reported that the inhibition of cathepsin S (*Ctss*) may suppress disease activity of SLE and lupus nephritis^27^. The deficiency of Blimp-1, transcriptional repressor of *Ctss*, in dendritic cells (DCs) led to increased *Ctss* expression, which resulted in a more diverse repertoire of follicular helper T cell (Tfh cell) contributing to autoimmunity in female C57BL/6 mice. A treatment of Blimp-1 knockout mice with *Ctss* inhibitor abolished the lupus-related phenotype and reduced the diversity of the Tfh cell TCR repertoire^28^. The elevated expression of Blimp-1 was induced by IL-12 in a STAT4-dependent manner and IL-10 expression in Th1 cell was up-regulated by Blimp-1^29^. The elevated Blimp-1 in SLE patients and in MRL mice positively correlated with the increase in plasma cells, autoantibodies and disease activity^30^. Although the functional role of *Ctss* in SLE is well-investigated, the information about *Ctse* in the pathogenesis of SLE is scarce. The functional roles of *Ctse* have been reported exclusively in regulatory T (Treg) cells. The elevation of *Ctse* gene was reported in IL-10/IL-35 double-deficient Treg cells, where Ctse enhanced TNF-related apoptosis-inducing ligand (TRAIL) expression and release of soluble form^31,32^, and activated CD4+ Foxp3+ Tregs and CD8+ TTregs utilize TRAIL as suppressive mechanisms by inducing apoptosis, promoting programmed regulated necrosis (necroptosis), and inhibiting cell proliferation^33^ to compensate for loss of IL-10 and IL-35^33^. Treg cells from *Il10* (−/−) mice, which fetal loss induced by LPS was at high frequency, were increased in pregnancy and the expression of *Ctse*, *Ifng*, and *Il12rb2* in Treg cells was also enhanced^34^. 293 T cells which were transfected with expression plasmids encoding TRAIL limited T cell proliferation, and was further enhanced in the presence of expression plasmids encoding *Ctse*^33^. This suggests that *Ctse* may play a role in enhancing the function of TRAIL by either increasing its activity via processing or increasing the generation of soluble TRAIL^35^. Expression of IL-10 and/or IL-35 by wild-type Tregs may suppress *Ctse* expression and thus reduce the contribution of TRAIL-mediated killing. Chloroquine, an anti-malarial drug, which has immune-modulating activity and lysosomotropic activity, enhanced TRAIL mediated apoptosis in cancer cells^36^. SLE patients had significantly greater expression of TRAIL, TNF-like weak inducer of apoptosis (TWEAK), and FasL on CD4+ T cells than healthy control, which correlated with disease activity^37^. It has been reported that increased T cell *Ctse* and subsequent TRAIL expression may exacerbate lupus by increasing CD4+ Th cell numbers and inhibiting CD8+ Tcytotoxic T cell-mediated killing of autoreactive B cells^38^ and it may not be a compensatory mechanism to limit lupus activities. In contrast to MRL mice, Tulone, C., *et al*. reported the natural *Ctse* deficiency in immune system of B6 mice because of a polymorphism of the promotor region of *Ctse* damped promotor activity of PU.1 binding consensus sequence in B6 mice. The deficiency is cell-type-specific, as protein levels in gut are not affected, while the deficiency affects B cell, T cells, macrophages and dendritic cells^39^. The reduction of *Ctse* expression is largely dependent on the genetic background of B6 mice rather than *Fas*^*lpr*^ mutation.

The role of IL-10 whether it promotes or limits disease activity of SLE remains controversial. Autoimmune lymphoproliferative syndrome (ALPS) is an inherited disorder in which genetic defects in proteins that mediate lymphocyte apoptosis, most often Fas, are associated with enlargement of lymph nodes and the spleen and a variety of autoimmune manifestations like MRL mice. ALPS patients have dramatic elevations in circulating IL-10, and double negative T cells (DNTC) are the dominant producers of IL-10 when assessed *ex vivo*^40^. Although immunosuppressive therapy of ALPS patients decreases DNTC and serum IL-10 levels^41^, the roles of DNTC and IL-10 remain unclear. IL-10/Janus kinase/signal transducer and activator of transcription 3 (STAT3) pathway drives the expression of Bim, anti-apoptotic Bcl-2 family member, in DNTC derived from ALPS patients^42^. The severity of lupus in MRL mice were enhanced by the deletion of *Il10* gene. It was associated with enhanced IFN-γ production by both CD4+ and CD8+ Tcells and increased serum concentration of IgG2a anti-dsDNA autoantibodies^43^. Administration of rIL-10 reduced IgG2a anti-dsDNA autoantibody production and Th1 cytokine responses in wild-type MRL-Fas(lpr) in early phase^43^. Although the beneficial role of IL-10 has been reported, anti-IL-10 antibody may have a beneficial effect at later phases of disease, because excessive amounts of IL-10 production may lead to enhanced autoantibody production and subsequent formation of pathogenic autoantibody-Ag complexes^44^.

In addition to IL-10-dependent mechanisms, *Ctse* plays another key role for the development of SLE. *Ctse* deficiency caused autophagy impairment concomitantly with increased damaged mitochondria as well as increased oxidative stress^45^. Aberrant autophagy played an important role in the development of SLE^46^. Gros *et al*. detected more autophagosomes in mice and human lupus T lymphocytes^47^. Chen, J., *et al*. found that increased autophagy in T cells from SLE patients due to energy “starvation”, excessively accumulated mitochondria and over-activated autophagy promoted apoptosis of T cells from SLE patients^48^. Since the accumulated defective mitochondria could not match the high energy consumption within T cells in lupus with high disease activity, they may thus induce apoptosis, subsequently increasing autoantibody formation^49^. Thus, the regulation of energy state of T cells could probably be a new treatment target for SLE. Chloroquine, which blocks lysosomal degradation, prevented the contents in autolysosome from recycling and was unable to maintain cellular energy levels. *Ctse* works for lysosomal degradation and relate to mitochondrial function such as intracellular ATP levels, mitochondrial membrane potential, and mitochondrial oxygen consumption^45^. Based on line of evidences, one can suppose that overexpression of *Ctse* may be compensatory mechanism acting to limit lupus severity, *Ctse* will suppress the increased autophagy via elevation of defective mitochondria in lupus.

In conclusion, we presented the evidence that hypomethylation and mutation of the mCGCG site of the *Ctse* intron 1 in MRL mice disrupt the ample binding of Kaiso which results in increased expression of *Ctse* and *Il10* in SLE T cells. Whether upregulation of *Ctse* and *Il10* is compensation or promoting factors for disease activity of SLE remains for future investigation.

## Methods

### Mice

Genetically lupus-prone female MRL/MpJ-*Fas*^*lpr*^/J (MRL) mice, B6.MRL-*Fas*^*lpr*^/J mice (B6MRL) (Jackson Laboratory), and C57BL/6 J (B6) mice (Charles River Laboratories) were purchased. At 16 weeks of age, MRL and B6 mice were sacrificed, and their spleen tissues were collected.

### Isolation of mouse primary T cells, B cells and peritoneal macrophages

Mouse splenic CD4+ T cells, CD8+ T cells, B cells were isolated using a CD4 isolation kit II (Miltenyi Biotec), CD8a isolation kit II (Miltenyi Biotec) and PanBcell isolation kit II (Miltenyi Biotec) by negative selection, respectively. A purity rate of >96.6% for isolated CD4+ T cells, CD8+ T cells and B cells were confirmed by flow cytometry. Peritoneal macrophages were obtained from B6 and MRL mice by washing their peritoneal cavities with 15 ml of ice-cold saline. The cells from individual mice were centrifuged at 1500 rpm for 5 minutes at 4 °C, washed in complete DMEM (Life Technologies) supplemented with 10% FBS (Life Technologies), 100 U/ml penicillin, and 100 μg/ml streptomycin, and subsequently adjusted to 1 × 10^7^ cells/ml. The cells were cultured on 12-well flat-bottom tissue culture plates (Corning) and incubated for 3 hours at 37 °C under 5% CO_2_ air.

### Genome wide DNA methylation and mRNA profiling using a next-generation sequencer

Genomic DNAs and total RNAs were purified from CD4+ T cells of MRL mice and B6 mice using All Prep DNA/RNA Mini Kit (Qiagen). The quality of the DNA was confirmed by fluorometer using Qubit dsDNA BR Assay Kits (Invitrogen). EpiXplore Methylated DNA Enrichment Kit (Clontech) was used for enriching methylated DNA fragments from the genomic DNA. First, we prewashed TALON Magnetic Beads and couple MBD2 protein to magnetic beads, bound methylated DNA to MBD2 protein/magnetic beads complexes, removed non- and hypomethylated DNA, eluted, and purified the methylated DNA. Illumina TruSeq ChIP Sample Prep Kit was used for the construction of sequencing libraries, and they were subjected to sequencing using Illumina HiSeq. The previously published mRNA sequencing data^10^ appeared in Gene Expression Omnibus (https://www.ncbi.nlm.nih.gov/geo/) under the accession number GSE87219 was analyzed with Cuffdiff program, and integrated with genome wide DNA methylation profiling data (GSE102421). In brief, significant methylation peaks in B6 and MRL mice were identified with model-based analysis of ChIP-Seq (MACS 1.4.2) using parameters bandwidth = 300, mfold = 10,30, and gzise = 1870000000^50^, mapped to genome, differentially methylated regions (DRMs) between B6 and MRL mice identified, and duplicate reads removed. The list of genes with read numbers of mRNA were merged with annotation of enriched regions. The read depth, enriched regions, mapping information was visualized by UCSC Genome Browser.

### EL4 cell line culture

The EL4 mouse T cell line was obtained from the American Type Culture Collection and cultured in DMEM (Life Technologies) supplemented with 10% FBS (Life Technologies), 100 U/ml penicillin, and 100 μg/ml streptomycin. A total of 4 × 10^6^ cells/ml (EL4 cells or MRL- and B6 mice-derived CD4+ T cells) were treated with 1.0 μM 5-azacytidine (5-azaC; Sigma-Aldrich) for 48 hours and with 30 nM Trichostatin A (TSA; WAKO) at 37 °C in a 5% CO_2_ incubator. The concentrations of 5-azaC and TSA were determined by the titration by counting Trypan Blue-negative viable cells. The percentages of viable cells were as follows; 5-azaC (93.3% at 0 μM, 89.6% at 0.2 μM, 82.9% at 1.0 μM, 0% at 5.0 and 10 μM) and TSA (97.5% at 0 nM, 85.4% at 30 nM, 49.6% at 300 nM, 44.6% at 1 μM, 38% at 3 μM).

### Bisulphite sequencing

Genomic DNA isolated from CD4+ T cells of MRL (n = 5) and B6 (n = 5) mice was bisulphite converted using the MethylEasy Xceed Rapid DNA Bisulphite Modification Kit (TaKaRa) following the manufacturer’s protocol. Cytosines are converted to uracils whereas 5-methylcytosines are unreactive. The 440 bp fragment within 583 bp region at intron 1 of *Ctse* was amplified by nested PCR with EpiTaq HS (TaKaRa) and a set of primers: Primer F1: 5′-TAGGTATGTTTTTTATTTATATTTTTAGTG-3′ and Primer R1: 5′-GACTAAATAAATTTTCTTTTTCTAATACTT-3′. PCR products were gel purified with NucleoSpinGel and PCR clean-up (TaKaRa) kits. DNA direct sequencing was performed with BigDye Terminator v3.1 Cycle Sequencing kit (Thermo Fisher Scientific) and methylation patterns at the level of individual CpG sites were examined.

Furthermore, the purified PCR products from CD4+ T cells of MRL (n = 1) and B6 (n = 1) mice were individually cloned into a T-vector pD20 using the DNA Ligation Kit Mighty Mix, and transformed to *E*.*coli* HST08 Premium Competent Cells (TaKaRa). The 72 plasmid clones in B6 and 65 in MRL were isolated and sequenced.

### Methylation specific PCR

To evaluate the methylation status of the CGCG motif in the *Ctse* promoter, genomic DNA (1 μg) from EL4 cells was purified and treated with *EcoR*I (TaKaRa) and the methylation-sensitive restriction enzyme *Acc*II (New England Biolabs). mCGCG is resistant to *Acc*II digestion, while CGCG is sensitive. After DNA re-purification, 50 ng of DNA was used as a template, and Primer F2: 5′-ACGGCTTTTGTGTTTTGGCT-3′ and Primer R2: 5′-ACATCAGCACTTAGGTTCTGCT-3′, or Primer R3: 5′-GCCCTGGTTCAATCCCTAAT-3′ were used for PCR. PCR products were electrophoresed on 2% agarose gels, visualized by ethidium bromide staining, and quantified with ImageQuant TL software (GE Health Life Sciences). The PCR products amplified with F2 and R2 corresponds to the amount of *Acc*II-resistant DNA, while the products by F2 and R3 reflects total amount of genomic DNAs.

### Patients and T lymphocyte purification

The 15 SLE patients (10 females and 5 males) who fulfilled at least 4 of the 11 revised criteria of the American College of Rheumatology for the classification of SLE^51^ enrolled and newly developed the disease. The samples were obtained in prior to the treatments with immunosuppressive agents and glucocorticoids. The disease activity was assessed by the SLE disease activity index (SLEDAI)^52^. The healthy volunteers were recruited as controls. The current clinical investigation was conducted in accordance with the ethical standards laid down in the Helsinki Declaration by the World Medical Association, as well as “Ethical Guidelines for Medical and Health Research Involving Human Subjects” presented by the Ministry of Health, Labour and Welfare. The studies were approved by the Ethical Committee, Okayama University Hospital (#1779). Written informed consents were obtained from all participants. CD4+ T lymphocytes were purified using the RosetteSep Human CD4+ TCD4+ T Cell Enrichment Cocktail (Stem Cell Technologies). The samples were centrifuged over a buoyant density medium, RosetteSep DM-L. The purified CD4+ TCD4+ T cells were recovered from the interface between the plasma and the buoyant density medium. Using flow cytometry, we established that the purified cells were >94% positive for CD4. Subsequently, both RNA and DNA were extracted from CD4+ T cells using the AllPrep DNA/RNA Mini Kit (Qiagen) according to the manufacturer’s protocol.

### RNA isolation and real-time RT-PCR

Total cellular RNAs from human and mouse samples were extracted with an RNeasy mini kit (Qiagen). cDNAs were reverse transcribed from mRNAs with a high-capacity cDNA RT kit (Thermo Fisher Scientific). Real-time PCRs for *Ctse* (Mm00456010_m1), *Il10* (Mm01288386_m1), *Il17a* (Mm00439618_m1), *Pdcd4* (Mm01266062_m1), *Gapdh* (Mm99999915_g1) for mouse, *CTSE* (Hs00157213_m1), *IL10* (Hs00961622_m1), *PDCD4* (Hs00377253_m1) and *GAPDH* (Hs02786624_g1) for human were performed using ABI TaqMan gene expression assays (Applied Biosystems) and normalized to GAPDH by the ΔΔCt method (Supplementary Table 4).

### Western blot analysis

The lysates of murine splenic CD4+ T cells were prepared by homogenization with CelLytic M, supplemented with a protease inhibitor cocktail (Sigma-Aldrich). Protein concentration was determined using the Pierce BCA protein Assay kit (Thermo Fisher Scientific). The samples were run on a 4–20% Mini-PROTEIN TGX Precast Gel (Bio-Rad) electrophoresis (SDS-PAGE) and blotted on Amersham Hybond P 0.45 µm PVDF (GE Healthcare Life Sciences). Primary antibodies against CTSE at 1:2,000 dilution (NB400-152, NOVUS) and beta-Actin at 1:1,000 dilution (ab8227; abcam) in 5% bovine serum albumin in TBS supplemented with 0.1% Tween 20 (TBS-T) were incubated overnight at 4 °C. Then, the blots were washed with TBS-T and incubated with donkey anti-rabbit IgG-HRP (1:5,000; SANTA CRUZ) for 45 minutes at room temperature. After three-time washes with TBS-T, bands were visualized with enhanced chemiluminescence using the Pirece Western Blotting Substrate Plus (Thermo Fisher Scientific). Protein bands were semiquantified by densitometry using ImageQuant TL software (GE Healthcare Life Sciences).

### Chromatin immunoprecipitation (ChIP)-qPCR

ChIP-qPCR was performed in accordance with the manufacturer’s instructions (Cell Signaling Technology). Briefly, cells were cross-linked with 1% formaldehyde. After the cross-linked cells were lysed, the chromatin was harvested and fragmented with enzymatic digestion using micrococcal nuclease. Nuclear extracts were further treated with an ultrasound sonicator with three sets of 20-s pulses. Immunoprecipitations were performed with Kaiso (ab12723; Abcam) and HDAC3 (ab7030; Abcam) antibodies. After DNA-protein cross-links were reversed, the DNA was purified, and qPCR was performed with Custom Taqman™ Gene Expression Assays (AIQJDG8) by forward primer: 5′-GCCTTCGGTGTGCATGCTA-3′ and reverse primer 5′-CACTTAGGTTCTGCTTATGGTAACGT-3, and probe 5′-TTTCCCCGCGAGACTT-3′ and all values were standardized with input DNA.

### ELISA

The amount of IL-10 protein secreted in the supernatants from cell cultures was measured by ELISA in accordance with the manufacturers’ instructions (mouse IL-10 ELISA Ready-SET-Go!, eBioscience). The absorbance was determined using a microplate reader set at 450 nm.

### Small Interfering RNAs (siRNAs) transfections

EL4 cells were transfected with 5 μM si-CTSE (Ctse Silencer Select Pre designed siRNA ID: s64606 and s64607) or si-Negative-Control (Si-Control) using Neon electroporation system (Thermo Fisher Scientific) in serum-free medium with one pulse with a voltage of 1080 Volts and width of 50 ms. Twenty-four hours after transfection, 10^6^ cells/ml were stimulated with 10 ng/ml phorbol 12-myristate 13-acetate (PMA) and 1 μM Ionomycin (Sigma-Aldrich).

### Electrophoretic mobility shift assay (EMSA)

Nuclear extracts were prepared from EL4 cells using NXTRACT (Sigma-Aldrich). In binding assays of 20 μl total volume, 28 μg of total nuclear extract from EL4 cells was incubated with 20 fmol of 3′ biotin-labeled oligonucleotide in the presence of 2 μl of 10× binding buffer (100 mM Tris, 500 mM KCl, 10 mM DTT [pH 7.5]) and 10 μM ZnSO4, 1 μl of 50% glycerol, 1 μg/μl poly(deoxyinosinic-deoxycytidylic) acid, 5 mmol/L MgCl_2_ and 1% Nonidet P-40 at room temperature for 20 minutes. The oligonucleotides and methylated oligonucleotides (eurofins) were annealed and used as probes or competitors (Supplementary Table 2). All competitors were used at 200-fold excess. For supershift experiments, the nuclear extracts were incubated with 4 μg of anti-Kaiso Ab (6 F/6F8, ab12723; Abcam) and 5 μg of anti- HDAC3 Ab (ab7030; Abcam). The binding mixtures were loaded onto 6% native acrylamide gel (Thermo Fisher Scientific) in Tris/borate/EDTA buffer and electrophoresed for 50 minutes at room temperature under a constant 100 V. The gels were then transferred to a nylon membrane at 4 °C for 50 minutes under a constant 100V and exposed to UV light to crosslink for 15 minutes. The DNA binding activity was detected using a LightShift chemiluminescent EMSA kit (Thermo Fisher Scientific). The image was obtained using an LAS-3000 IR.

### Construction of reporter plasmids

The Kaiso regulatory region in *Ctse* gene was amplified from B6 or MRL mice genomic DNAs isolated from splenic CD4+ T cells by using 5′-primer with *Xho*I restriction site (5′-GGGGGGCTCGAGGGACATCCTTCTAGGAAGCG-3′), and 3′-primer with *Hind*III restriction site (5′-GGGGGGAAGCTTGGTGTGTGTGTGTCTGATACTG-3′), and TaKaRa Taq Hot Start Version. The PU.1 promoter region^17^ in *Ctse* gene was amplified with 5′-primer with *Kpn*I site (5′-GGGGGGGTACCGGACCCTCATTCACTTTTGC-3′) and 3′-primer with *Xho*I site (5′-GGGGGCTCGAGGAGACAGGGCCTTACCTGTG-3′). The PCR products of Kaiso regulatory region and PU.1 promoter region were ligated to multiple cloning site of the pGL4.10 [*luc2*] Vector (Promega)(Supplementary Fig. 2).

Plasmids were transformed in TOP10 (Invitrogen) expressing Dam and Dcm methylases, which methylate GATC in the former, and CCAGG and CCTGG in the latter. Thus, CGCG and CGGG sites were not methylated in TOP10 competent cells. Twenty micrograms of plasmids were methylated using 16 U of CpG methyltransferase (*M*. *SssI*) and S-adenosyl methionine (SAM: New England Biolabs) at 37 °C for 16 hours, with subsequent inactivation of enzyme at 65 °C for 20 minutes. Mock-methylation reactions were also performed in the absence of *M*. *SssI* and SAM. The methylated or mock-methylated constructs were purified using EndoFree Plasmid Maxi Kit (Qiagen) and the methylation status of each construct was determined by methylation-sensitive restriction enzyme *Acc*II (TaKaRa) for plasmid constructs and empty vector.

### Cell transfection

The transfection of plasmids into EL4 cells was performed using Neon electroporation system (Thermo Fisher Scientific) in serum-free medium with one pulse with a voltage of 1080 Volts and width of 50 ms. pGL4.10 [*luc2*] derived constructs (Supplementary Fig. 2). Following the transfection, 2 × 10^5^ EL4 cells were cultured in 24-well plates and harvested after 24-hour incubation. Cytoplasmic extracts were prepared using a luciferase assay kit (Promega) in accordance with the manufacturer’s instructions.

### Statistical analyses

All results are shown as the mean ± standard deviation (SD) of data from at least three separate experiments, each performed with more than triplicate samples. The differences between the groups were analyzed using ANOVA with a Tukey–Kramer post hoc test or an unpaired *t* test, when appropriate, to determine the differences. P values less than 0.05 were considered significant. All statistical analyses were performed using the JMP 11.2.0 software package (SAS Institute).

### Ethical approval and informed consent

The experiments were approved by the Animal Care and Use Committee of the Department of Animal Resources, Advanced Science Research Center, Okayama University (OKU-2015569 and OKU-2013092). All animal experiments were performed in accordance with relevant guideline and regulations.

## Supplementary information

Supplementary Tables and Figures
