# Supplementary material for: Regulation of Cathepsin E gene expression by the transcription factor Kaiso in MRL/lpr mice derived CD4+ T cells
Source: Sci Rep. 2019 Feb 28;9:3054. doi: 10.1038/s41598-019-38809-y (PMC6395770; doi:10.1038/s41598-019-38809-y)
Supplement: Supplementary file 1 — Supplementary Tables and Figures [file 41598_2019_38809_MOESM1_ESM.pdf]

Supplementary information

Regulation of Cathepsin E gene expression by the transcription factor Kaiso in MRL/lpr mice derived CD4+ T cells

Sumie Hiramatsu, M.D., Katsue S Watanabe, M.D., Ph.D., Sonia Zeggar, M.D., Yosuke Asano, M.D., Yoshia Miyawaki, M.D., Ph.D., Yuriko Yamamura, M.D., Ph.D., Eri Katsuyama, M.D., Ph.D., Takayuki Katsuyama, M.D., Ph.D., Haruki Watanabe, M.D., Ph.D., Mariko Takano-Narazaki, M.D., Ph.D., Yoshinori Matsumoto, M.D., Ph.D., Tomoko Kawabata, M.D., Ph.D., Ken-Ei Sada, M.D., Ph.D., and Jun Wada M.D., Ph.D.

Supplementary Table 1 Genome-wide DNA methylation and mRNA profiling in splenic CD4+ T cells purified in MRL/lpr lupus-prone mice (MRL) and C57BL/6 (B6) mice

RNA sequencing					DNA Methylation Analysis by sequencing					
Official Gene Symbol	FPKM[1] (B6)	FPKM[1] (MRL)	Fold change[2]	P value	Position	Length	Summit	Tags	-10log10 (P value)	Fold enrichment[3]
Ctse	8.654	172.194	4.31449	7.05×10^{-7}	Chr 1: 131641487-131642069	583	265	37	50.8	43.6
Col6a4	0.004	1.052	8.04371	1.09×10^{-5}	Chr 9: 106028814-106029450	637	219	20	50.83	101.91
Rapgef4	9.896	0.380	-4.70231	5.06×10^{-5}	Chr 2: 72238362-72239704	1343	785	33	71.26	113.9
Nrg1	0.031	2.654	6.44407	9.77×10^{-5}	Chr 8: 31892346-31893371	1026	587	34	82.06	185.84
Fam135a	0.032	1.323	5.35945	0.000122	Chr 1: 24001838-24002475	638	437	25	64.12	102.56
Sort1	0.497	8.963	4.1715	0.000147	Chr 3: 108301724-108302119	396	198	15	50.09	64.1
Trim30d	5.199	0.226	-4.52563	0.000303	Chr 7: 104514606-104515847	1242	717	26	53.69	77.93

1. FPKM：Reads per kilobase of exon model per million mapped reads.

2. Fold change = $\log2(FPKM_{MRL}/FPKM_{B6})$

3. Fold enrichment: The rate of the peak of the apex when compared with the background.

Supplementary Table 2 EMSA probes of CGCG motif in Kaiso regulatory region C57BL/6J (B6) and MRL/MpJ-*Fas*^{lpr}/J (MRL) mice

EMSA probes	Sequences
B6-Methylated probe (B6-Me)	5′-CATGTGTTTTCCCmCGmCGAGACTTACCTCTA-3′<br>3′-GTACACAAAAGGGGmCGmCTCTGAATGGAGAT-5′
MRL probe (MRL)	5′-CATGTGTTTTCCCCGGGAGACTTACCTCTA-3′<br>3′-GTACACAAGAGGGGCCCTCTGAATGGAGAT-5′
MRL-Methylated probe (MRL-Me)	5′-CATGTGTTTTCCCmCGGGAGACTTACCTCTA-3′<br>3′-GTACACAAGAGGGGCCmCTCTGAATGGAGAT-5′

Supplementary Table 3 Patient demographics.

Parameters	SLE (n=15)<br>Mean±standard deviation
Sex (male / female)	5/10
Race	Japanese
Age (years)	46±15.1
SLEDAI (systemic erythematosus disease activity index)	17.1±8.5
BILAG (British Isles Lupus Assessment Group disease activity index)	14.5±4.9
Anti-nuclear antibody (ANA) positive (%)	93.3
ANA titer (x)	828.6±638.3
dsDNA antibody positive (%)	86.7
Anti-dsDNA antibody (IU/mL)	134.4±144.0
Anti-Sm positive (%)	26.7
Anti-Sm antibody (U/ml)	20.4±42.4
Anti-ribonucleoprotein (RNP) positive (%)	40.0
Anti-RNP antibody (U/mL)	81.6±108.9
Anti-cardiolipin (CL) antibody (%)	33.3
Anti-CL antibody (U/mL)	18.4±28.4
Aniti-β2 glycoprotein I (β2GPI) positive (%)	6.7
Aniti-β2GPI antibody (U/mL)	3.8±9.9
Lupus anticoagulant positive (%)	40.0
Rheumatoid factor (RF) (IU/mL)	11.2±11.6
Anti-SS-A positive (%)	80.0
Anti-SS-A antibody (U/mL)	159.7±105.2
Anti-SS-B positive rate (%)	13.3
Anti-SS-B antibody (U/mL)	16.0±44.2
IgG (mg/dL)	2255.6±504.3
IgA (mg/dL)	338.1±145.9
IgM (mg/dL)	161.7±131.0
C3 below normal range (%)	80.0
C3 titer (mg/dL)	44.6±20.6
C4 below normal range (%)	93.3
C4 titer (mg/dL)	7.0±4.2
CH50 below normal range (%)	93.3
CH50 (U/mL)	17.2±10.7
Serum creatinine (mg/dL)	0.7±0.2
Urine protein / creatinine (g/gCr)	2.1±3.9
White blood cells ($\times 10^3/\mu L$)	3304.0±1531.6
Lymphocytes ($\times 10^3/\mu L$)	861.3±1037.0
Hemoglobin (g/dL)	11.3±1.8
Platelets ($\times 10^4/\mu L$)	16.0±7.1
Lupus nephritis (%)	40.0
Arthritis (%)	66.7
Skin rash (%)	73.3
Neuropsychiatric SLE (%)	20.0

Supplementary Table 4 The list of the primers used for RT-PCR. Genes analyzed by Taqman Gene Expression Assay.

Gene Name	Probe Number
Ctse	Mm00456010_m1
Il10	Mm01288386_m1
Il17a	Mm00439618_m1
Pdcd4	Mm01266062_m1
Gapdh	Mm99999915_g1
CTSE	Hs00157213_m1
IL10	Hs00961622_m1
PDCD4	Hs00377253_m1
GAPDH	Hs02786624_g1

Supplementary Figure 1

Figure 1B Original figure

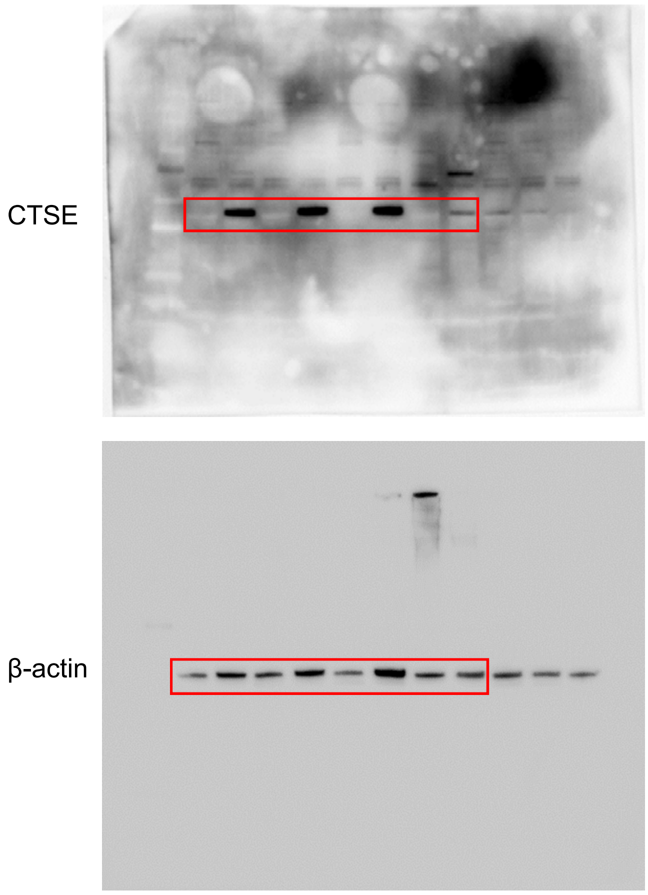

Figure 2A Original figure

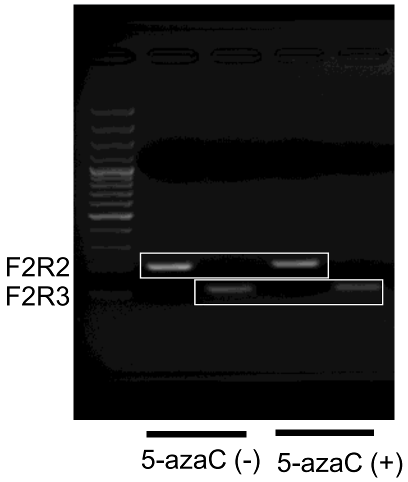

Figure 2D Original figure

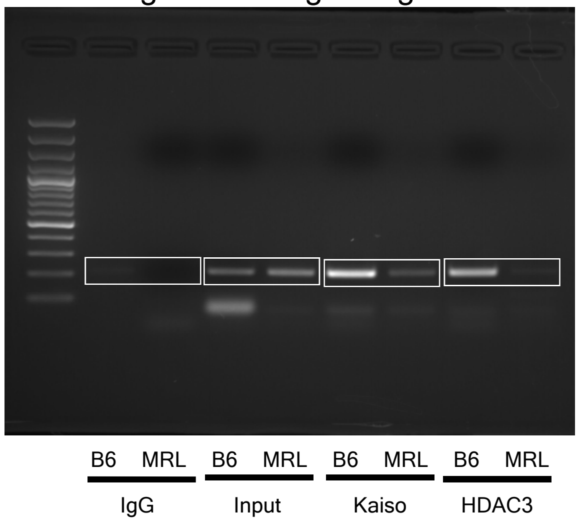

Figure 2C Original figure

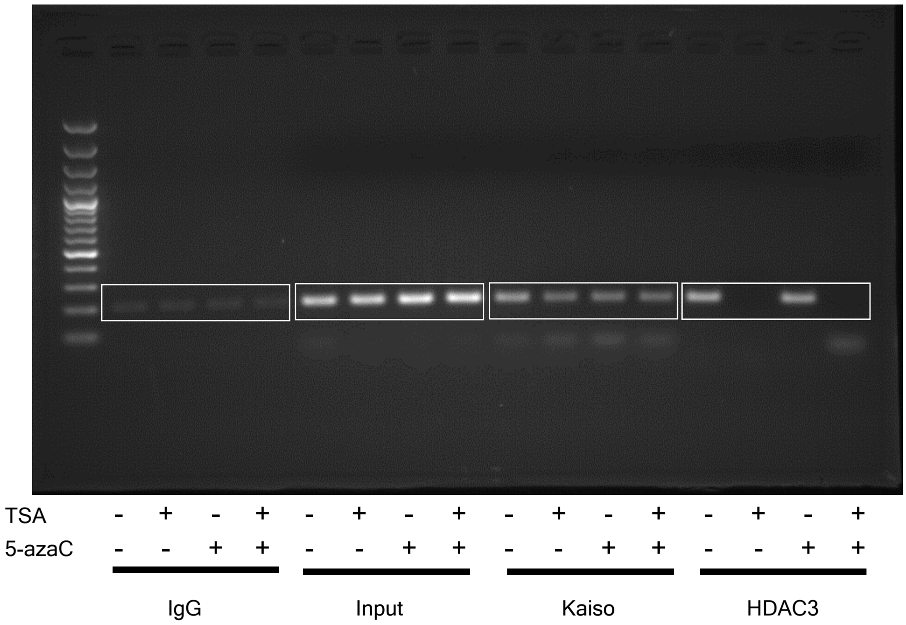

Supplementary Figure 1
Uncropped blot images of Western blot in Figure 1B, and agarose gel electrophoresis in Figures 2A, 2C and 2D.

Supplementary Figure 2

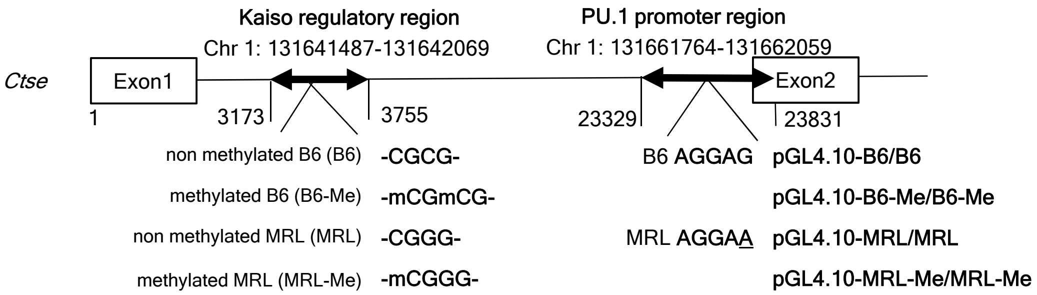

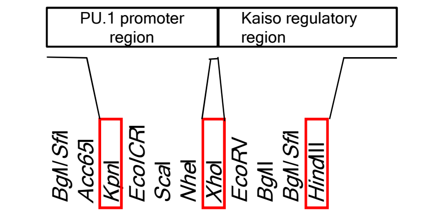

Multiple cloning site of pGL4.10 [*luc2*] Vector

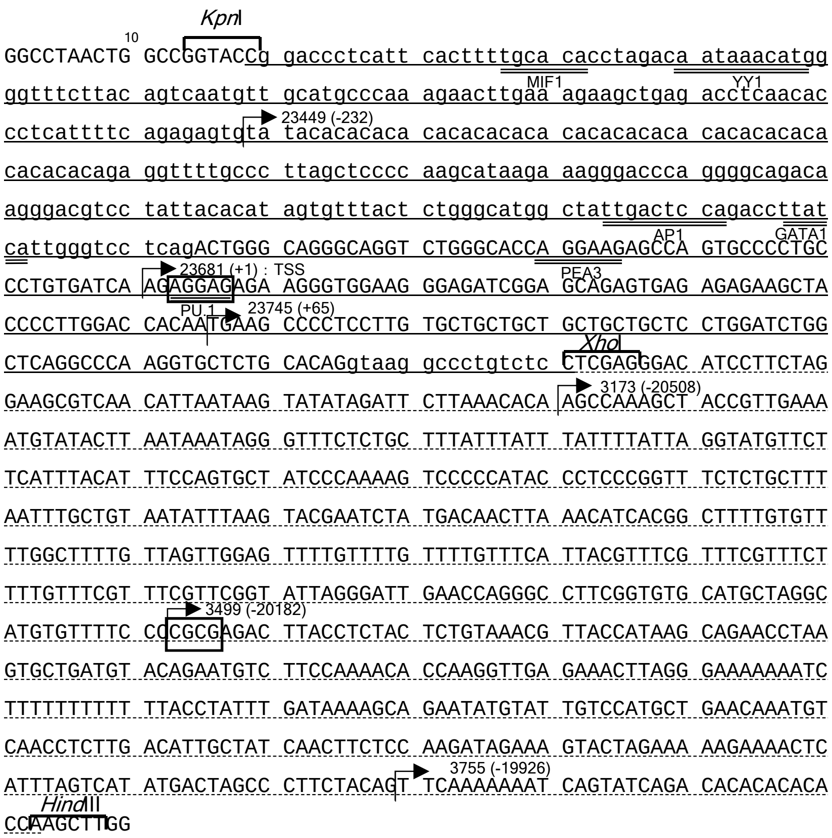

Supplementary Figure 2

Preparation of pGL4.10 [*luc2*] vector constructs. The PCR products of Kaiso regulatory region (3173-3755) and PU.1 promoter region (23329-23831) were ligated to multiple cloning site of the pGL4.10 [*luc2*] Vector (Promega). Abbreviations: AP1; Jun proto-oncogene, AP-1 transcription factor subunit; GATA1, GATA binding protein 1; Kaiso, zinc finger and BTB domain containing 33; MIF1, predicted gene 4924 (mouse inhibitor of hour 1); PEA3, Phosphatidylinositol-4-phosphate 5-kinase and related FYVE finger-containing proteins signal transduction mechanisms; PU.1, spleen focus forming virus (SFFV) proviral integration oncogene; YY1, YY1 transcription factor.

Supplementary Figure 3

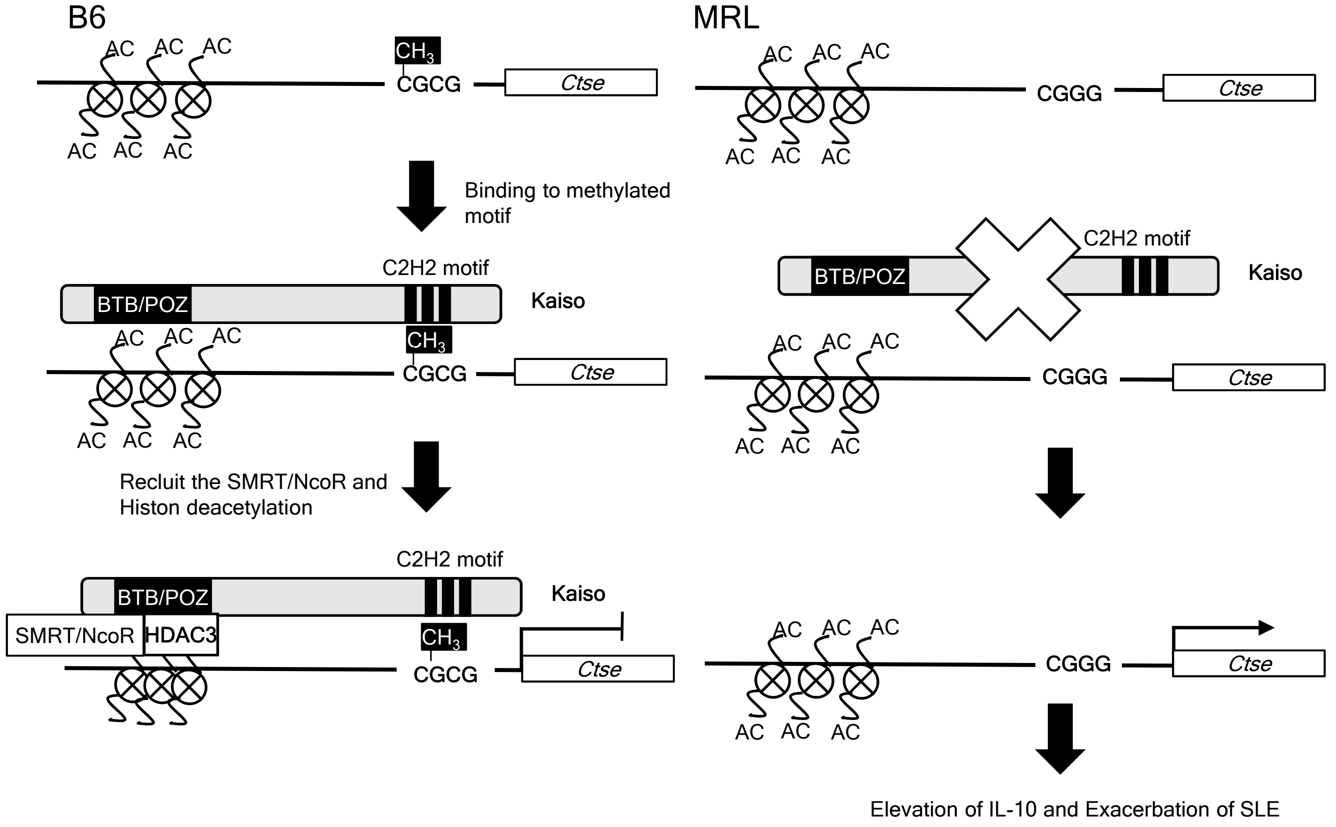

Supplementary Figure 3

Schematic models of *Ctse* transcriptional regulation by Kaiso and HDAC3. Hypomethylated and mutated mCGCG motif to CGGG negatively regulates the recruitment of suppressive transcription factor complex, Kaiso and HDAC3, and promotes IL-10 production in T cells from MRL mice.
